# Account *of the* Medical Effects *of* Electricity

**Published:** 1814-01

**Authors:** Parker Cleveland

**Affiliations:** Professor of Mathematics and Natural Philosophy in Bowdoin College


*For the Medical and Physical Journal.*

ACCOUNT *of the* MEDICAL EFFECTS *of* ELECTRICITY, *by* PARKER CLEVELAND, ESQ. *Professor of Mathematics and Natural Philosophy in Bowdoin College.*

IT is not in the least surprising, that an agent so subtle and powerful as electricity, and so wonderful in its effects, should have excited much speculation, and led to many fanciful conjectures with regard to its influence, as a remedy in disease. Not content that it should occupy its proper rank, its friends have, in some periods, gradually extended its supposed efficacy to almost every disease incident to the human system. But, whatever may have been the varying reputation of electricity, in regard to its medical application, sufficient is now known to authorise the belief, that it is a powerful agent in the animal economy, and exerts an important influence on the human body, both in a healthy and diseased state. It may yet prove to be the principal agent in the secretions, compositions, and decompositions of both the animal and vegetable kingdoms. One thing is certain; the only method of ascertaining its effects on the human system, is by careful experiment, accurate observation, and a numerous collection of facts. The discovery of galvanism can hardly fail of promoting these objects, by the facilities it presents for electrical inquiry.

The following cases may not contain any thing hitherto unobserved, but insulated facts, when collected and compared, may hereafter lead to some important general deductions.

In April last, Mr. P. of Bath, called on me for the purpose of receiving electricity. He was entirely deprived of the power of speech, and unable to produce any distinct sound.

---

With

With a great and painful effort he succeeded in making known his wishes by an imperfect whisper. When he thus whispered, both the shoulders were considerably raised. He complained of numbness and stricture in the breast. In this situation, Mr. P. had continued without any relief for about six weeks. His general health was comfortable. At first I permitted him to receive several shocks from a Leyden jar, of the capacity of one pint, about half charged, causing the fluid to pass from one hand to the other through the arms and breast. This charge he did not appear to feel above the wrists. I then gave the jar very nearly its full charge, and passed it two or three times through the breast. He then spoke, in a very audible manner, saying "my voice is coming." His voice then resembled that of a man somewhat hoarse with a cold. He added, "I feel a little of it (meaning the numbness) yet about the lower part of my breast." I then passed two or three similar charges through the part in which the numbness was felt. By this time Mr. P. spoke with his natural voice, and, as he assured me, with perfect ease; being entirely free from any numbness. As he was leaving the laboratory, I observed that the finger which he had applied to the brass knob of the jar was entirely devoid of colour. It was cold and incapable of sensation. I passed a few charges through it, and in the course of a few minutes its colour returned.

So great a change in the space of five minutes was to me surprising; and I took the liberty of requesting Dr. Stockbridge of Bath, whose patient Mr. P. was, and who had sent him to me for the aforesaid purpose, to furnish me with a history of this man. Dr. S. very politely forwarded me an account of his patient; of which the following is an abstract. In October, 1787, Mr. P. (being then eighteen years old) while making uncommon efforts to lift a boat, felt something give way in his back. He immediately fell to the ground, and there remained several minutes, in great pain, with fainting. After recovering so much as to walk, he waded through water, as deep as his breast, and received a severe cold. In about a week he was attacked with a fever, attended with acute pain in the left side of the throat, and a severe cough without expectoration. His fever soon left him, but he remained infirm about five years, and was supposed by his physician to be consumptive. On the expiration of this time he so far recovered his health, as to be able to attend to his usual business. He was, however, soon visited by another disease. This commenced with a trembling of the extremities, and an indescribable uneasiness in the feet and hands, extending in a few minutes to the breast, and causing him instantly instantly to fall senseless and convulsed. After remaining from two to five minutes, he recovered, and felt no inconvenience, excepting some degree of debility. He was frequently attacked in the same way, and generally after great fatigue and exposure to cold.

Mr. P. is a tanner. In June, 1796, while shaving leather at the currying beam, he was seized with acute pain at the point of the sternum and short ribs. This suddenly extended up the breast to the throat, and instantly subsided, leaving him entirely deprived of the power of speech, with a sensation of numbness and stricture across the lungs. The power of coughing was almost suspended, and it was with difficulty and a painful sense of suffocation, that any mucus collected on the lungs, could be expectorated. In this state he remained eighteen days, his general health being good, and sleep easy. He was at this time relieved by a few shocks of electricity, passed through the chest. Mr. P. enjoyed comfortable health, till September 1802, when he was again deprived of the power of speech. He complained of numbness and stricture on each lobe of the lungs, while in the region of the sternum he felt free and natural. At this time he applied to Dr. S. who advised an epispastic, and a free use of onions, garlic, &c. By feeding on these, he found himself relieved without employing the blister. He continued subject to slight returns of this disease, until 1806, when a large abscess, formed on the back between the scapula and spine, suppurated and discharged copiously; after which he was entirely free from any attack, and enjoyed good health till the last spring, when he was relieved in the manner before related. A short time since I understood he continued in the possession of comfortable health.

Another case, in which the subject was deprived of the power of speech, and in which electricity was applied without success, was that of a child, aged about ten years. While engaged in play, one of his companions threw a stone, which struck him on the back of the neck; in consequence of which blow, the child instantly became unable to speak, and his tongue remained incapable of motion, being very much withdrawn from the mouth toward the throat. In about half an hour he so far recovered his speech, as to inform his parents in what manner he had been injured. He then relapsed, and remained incapable of speaking for two months. On application to Dr. Lincoln, of Topsham, he advised to employ electricity. The child was accordingly brought to me for that purpose; but, so great was his terror, I was unable to pass any shocks through the neck. I could do no more than fill the child with the fluid, and draw a few sparks through through flannel from the neck. No good effect followed. Dr. L. then applied a blister, which afforded immediate relief.

A short time after, the same child, falling backward from his seat at school, and striking the same part of his neck, where he had received the blow from the stone, was again deprived of the power of speech. In this situation his friends permitted him to remain for five years, without causing any application to be made. At the expiration of this period, the boy was engaged in a violent scuffle, in which his limbs were very much exerted, and his passions somewhat excited. During the ensuing night he awoke from sleep, found his tongue at command, and the power of speech returned. Very possibly electricity might have given relief in the latter case, could it have been communicated in shocks. This, however, cannot be inferred from the former case, as the loss of speech in the two instances, was attended by very different circumstances.—*New England Journal of Medicine and Surgery, Jan.* 1813.

